# Ethyl 2-amino-4-(3-chloro­phen­yl)-5,10-dioxo-5,10-dihydro-4*H*-benzo[*g*]chromene-3-carboxyl­ate

**DOI:** 10.1107/S160053680901753X

**Published:** 2009-05-20

**Authors:** Xiao Hu, Song Lei, Chang-Sheng Yao

**Affiliations:** a Xuzhou Ruisai Technology Industry Co. Ltd, Xuzhou 221004, People’s Republic of China; b Department of Chemistry, Xuzhou Normal University, Xuzhou 221116, People’s Republic of China; c Key Laboratory of Biotechnology for Medical Plants of Jiangsu Province, Xuzhou 221116, People’s Republic of China

## Abstract

The title mol­ecule, C_22_H_16_ClNO_5_, was obtained by the reaction of (*E*)-ethyl 3-(3-chloro­phen­yl)-2-cyano­acrylate and 2-hydroxy­naphthalene-1,4-dione catalysed by triethylamine in ethanol. In the crystal structure, the chlorobenzene ring makes a dihedral angle of 88.63 (4)° with the fused ring system. The six-membered ring formed by an intra­molecular N—H⋯O hydrogen bond is almost planar. The crystal packing is stabilized by N—H⋯O hydrogen bonds.

## Related literature

For the anti­tumor activity of 4*H*-naphtho[2,3-*b*]pyran-5,10-dione derivatives, see: Fujimoto (2007 ▶); Perchellet *et al.* (2001 ▶); Zhan *et al.* (2007 ▶). For natural products containing *H*-naphtho[2,3-*b*]pyran-5,10-dione, see: Jassbi *et al.* (2004 ▶); Rodriguez *et al*. (2003 ▶).
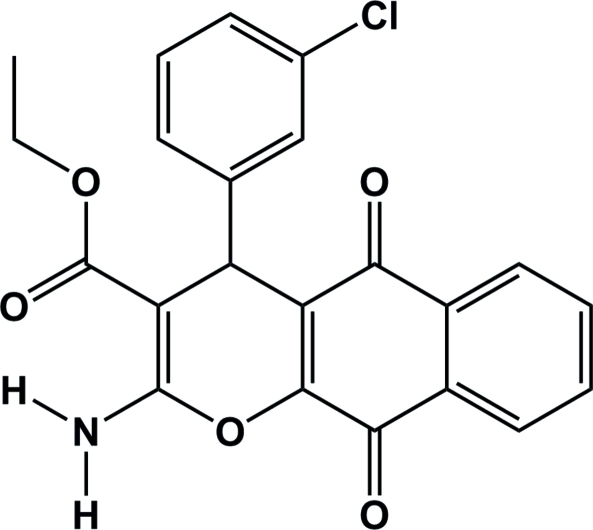


## Experimental

### 

#### Crystal data

C_22_H_16_ClNO_5_
*M* *_r_* = 409.81
Triclinic, 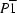

*a* = 6.1175 (17) Å
*b* = 10.021 (3) Å
*c* = 15.967 (5) Å
α = 84.840 (13)°
β = 87.714 (12)°
γ = 67.429 (8)°
*V* = 900.2 (4) Å^3^
*Z* = 2
Mo *K*α radiation
μ = 0.25 mm^−1^
*T* = 113 K
0.32 × 0.30 × 0.20 mm

#### Data collection

Rigaku Saturn diffractometer
Absorption correction: multi-scan (Jacobson, 1998 ▶) *T*_min_ = 0.924, *T*_max_ = 0.952
11338 measured reflections
4261 independent reflections
3031 reflections with *I* > 2σ(*I*)
*R*_int_ = 0.033

#### Refinement

*R*[*F* ^2^ > 2σ(*F* ^2^)] = 0.034
*wR*(*F* ^2^) = 0.097
*S* = 1.01
4261 reflections
272 parameters
H atoms treated by a mixture of independent and constrained refinement
Δρ_max_ = 0.37 e Å^−3^
Δρ_min_ = −0.45 e Å^−3^

### 

Data collection: *CrystalClear* (Rigaku/MSC, 2002 ▶); cell refinement: *CrystalClear*; data reduction: *CrystalClear*; program(s) used to solve structure: *SHELXS97* (Sheldrick, 2008 ▶); program(s) used to refine structure: *SHELXL97* (Sheldrick, 2008 ▶); molecular graphics: *SHELXTL* (Sheldrick, 2008 ▶); software used to prepare material for publication: *SHELXTL*.

## Supplementary Material

Crystal structure: contains datablocks global, I. DOI: 10.1107/S160053680901753X/hg2512sup1.cif

Structure factors: contains datablocks I. DOI: 10.1107/S160053680901753X/hg2512Isup2.hkl

Additional supplementary materials:  crystallographic information; 3D view; checkCIF report

## Figures and Tables

**Table 1:** Hydrogen-bond geometry (Å, °)

*D*—H⋯*A*	*D*—H	H⋯*A*	*D*⋯*A*	*D*—H⋯*A*
N1—H1⋯O4	0.898 (18)	2.049 (18)	2.6827 (17)	126.5 (15)
N1—H2⋯O2^i^	0.880 (19)	2.12 (2)	2.9913 (17)	170.2 (18)

Symmetry code: (i) 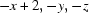.

**Fig. 1.**
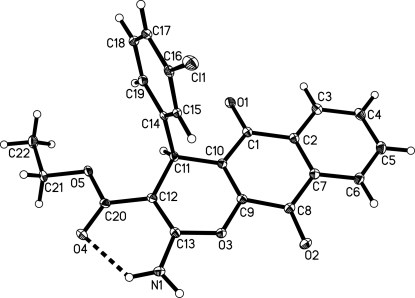
The structure of (I), showing 30% probability displacement ellipsoids and the atom-numbering scheme.

**Fig. 2.**
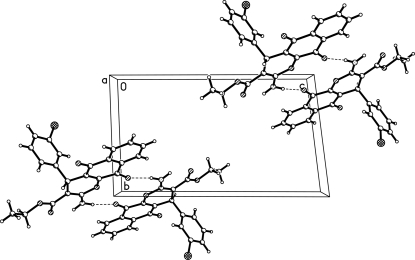
The packing diagram of (I). Intermolecular hydrogen bonds are shown as dashed lines.

## supplementary crystallographic information

### Comment

The derivatives of 4*H*-naphtho[2,3-*b*]pyran-5,10-dione have antitumor activities (Fujimoto, 2007; Zhan *et al.*, 2007; Perchellet *et al.*, 2001). Besides, some natural products also contain this moiety (Rodriguez *et al.*, 2003; Jassbi *et al.*, 2004). In order to develop new potential antitumor chemicals, a series of novel 4*H*-naphtho[2,3-*b*]pyran-5,10-dione derivatives based on the scaffolds of natural products have been synthesized. However, to the best of our knowledge, there are no reports on the crystal structure of these compounds. Determination of the molecular structure is crucial to the study of the structure and activity relationship. Here we report the crystal structure of the title compound, (I).

The molecular structure of (I) is shown in Fig. 1. It consists of five rings, considering the six-membered ring formed by the intramolecular N1—H1···O4 hydrogen bond (Table 1). The dihedral angles between the neighbouring rings show that the naphthalene ring and the pyran ring in an envelope conformation are almost coplanar. The phenyl ring bonded to the pyrans ring is almost perpendicular to the fused ring, for the dihedral angle is 88.63 (4)°. In the molecular structure, the crystal packing is stabilized N1—H2···O2 intermolecular hydrogen bonds. (Figs.2, Table 1)

### Experimental

The title compound was synthesized by the reaction of (*E*)-ethyl 3-(3-chlorophenyl)-2-cyanoacrylate (1 mmol) and 2-hydroxynaphthalene-1,4-dione (1 mmol) catalyzed by Et_3_N in 15 ml ethanol at reluxing temperature. After cooling, the solvent was removed at reduced pressure and the residue was washed with water and recrystallized from ethanol, which gave single crystals suitable for X-ray diffraction.

### Refinement

The hydrogen atoms bonded to nitrogen atom was positioned from a Fourier difference map and were refined freely. Other H atoms were placed in calculated positions, with C—H = 0.95-1.00 Å, and included in the final cycles of refinement using a riding model, with *U*_iso_(H) = 1.2*U*_eq_ (parent atom).

### Crystal data

**Table:**

C_22_H_16_ClNO_5_	*Z* = 2
*M**_r_* = 409.81	*F*(000) = 424
Triclinic, *P*1	*D*_x_ = 1.512 Mg m^−^^3^
Hall symbol: -P 1	Mo *K*α radiation, λ = 0.71070 Å
*a* = 6.1175 (17) Å	Cell parameters from 2873 reflections
*b* = 10.021 (3) Å	θ = 2.2–27.9°
*c* = 15.967 (5) Å	µ = 0.25 mm^−^^1^
α = 84.840 (13)°	*T* = 113 K
β = 87.714 (12)°	Block, red
γ = 67.429 (8)°	0.32 × 0.30 × 0.20 mm
*V* = 900.2 (4) Å^3^	

### Data collection

**Table:**

Rigaku Saturn diffractometer	4261 independent reflections
Radiation source: rotating anode	3031 reflections with *I* > 2σ(*I*)
confocal	*R*_int_ = 0.033
Detector resolution: 7.31 pixels mm^-1^	θ_max_ = 27.9°, θ_min_ = 2.2°
ω scans	*h* = −8→8
Absorption correction: multi-scan (Jacobson, 1998)	*k* = −13→13
*T*_min_ = 0.924, *T*_max_ = 0.952	*l* = −20→20
11338 measured reflections	

### Refinement

**Table:**

Refinement on *F*^2^	Secondary atom site location: difference Fourier map
Least-squares matrix: full	Hydrogen site location: inferred from neighbouring sites
*R*[*F*^2^ > 2σ(*F*^2^)] = 0.034	H atoms treated by a mixture of independent and constrained refinement
*wR*(*F*^2^) = 0.097	*w* = 1/[σ^2^(*F*_o_^2^) + (0.0583*P*)^2^] where *P* = (*F*_o_^2^ + 2*F*_c_^2^)/3
*S* = 1.01	(Δ/σ)_max_ < 0.001
4261 reflections	Δρ_max_ = 0.37 e Å^−^^3^
272 parameters	Δρ_min_ = −0.45 e Å^−^^3^
0 restraints	Extinction correction: *SHELXL97* (Sheldrick, 2008), Fc^*^=kFc[1+0.001xFc^2^λ^3^/sin(2θ)]^-1/4^
Primary atom site location: structure-invariant direct methods	Extinction coefficient: 0.020 (4)

### Special details

**Table:**

Geometry. All e.s.d.'s (except the e.s.d. in the dihedral angle between two l.s. planes) are estimated using the full covariance matrix. The cell e.s.d.'s are taken into account individually in the estimation of e.s.d.'s in distances, angles and torsion angles; correlations between e.s.d.'s in cell parameters are only used when they are defined by crystal symmetry. An approximate (isotropic) treatment of cell e.s.d.'s is used for estimating e.s.d.'s involving l.s. planes.
Refinement. Refinement of *F*^2^ against ALL reflections. The weighted *R*-factor *wR* and goodness of fit *S* are based on *F*^2^, conventional *R*-factors *R* are based on *F*, with *F* set to zero for negative *F*^2^. The threshold expression of *F*^2^ > σ(*F*^2^) is used only for calculating *R*-factors(gt) *etc*. and is not relevant to the choice of reflections for refinement. *R*-factors based on *F*^2^ are statistically about twice as large as those based on *F*, and *R*- factors based on ALL data will be even larger.

### Fractional atomic coordinates and isotropic or equivalent isotropic displacement parameters (Å^2^)

**Table:**

	*x*	*y*	*z*	*U*_iso_*/*U*_eq_	
Cl1	0.49377 (7)	0.57379 (4)	0.30597 (2)	0.02735 (12)	
O1	−0.07224 (16)	0.26225 (11)	0.15817 (6)	0.0199 (2)	
O2	0.68318 (17)	0.12419 (11)	−0.04552 (6)	0.0231 (2)	
O3	0.75549 (16)	0.04439 (10)	0.11440 (5)	0.0172 (2)	
O4	0.88099 (17)	−0.17355 (11)	0.35482 (6)	0.0225 (2)	
O5	0.48890 (16)	−0.07946 (10)	0.38009 (6)	0.0184 (2)	
N1	1.0294 (2)	−0.10163 (13)	0.20348 (8)	0.0191 (3)	
C1	0.0990 (2)	0.23745 (14)	0.11164 (8)	0.0156 (3)	
C2	0.0730 (2)	0.29181 (14)	0.02047 (8)	0.0162 (3)	
C3	−0.1499 (2)	0.37702 (15)	−0.01112 (8)	0.0194 (3)	
H3	−0.2854	0.3985	0.0244	0.023*	
C4	−0.1737 (3)	0.43104 (16)	−0.09541 (9)	0.0223 (3)	
H4	−0.3264	0.4885	−0.1173	0.027*	
C5	0.0222 (3)	0.40187 (16)	−0.14729 (9)	0.0228 (3)	
H5	0.0044	0.4412	−0.2042	0.027*	
C6	0.2449 (3)	0.31522 (15)	−0.11653 (8)	0.0203 (3)	
H6	0.3795	0.2939	−0.1524	0.024*	
C7	0.2707 (2)	0.25930 (14)	−0.03244 (8)	0.0167 (3)	
C8	0.5078 (2)	0.16626 (14)	−0.00028 (8)	0.0164 (3)	
C9	0.5273 (2)	0.12298 (14)	0.09172 (8)	0.0155 (3)	
C10	0.3409 (2)	0.15414 (14)	0.14472 (8)	0.0147 (3)	
C11	0.3707 (2)	0.10501 (14)	0.23718 (8)	0.0148 (3)	
H11	0.2601	0.0547	0.2528	0.018*	
C12	0.6221 (2)	−0.00336 (14)	0.25298 (8)	0.0155 (3)	
C13	0.7982 (2)	−0.02151 (14)	0.19472 (8)	0.0157 (3)	
C14	0.3041 (2)	0.23611 (14)	0.28925 (8)	0.0145 (3)	
C15	0.4265 (2)	0.32887 (14)	0.27878 (8)	0.0155 (3)	
H15	0.5580	0.3086	0.2414	0.019*	
C16	0.3534 (2)	0.45117 (15)	0.32365 (8)	0.0191 (3)	
C17	0.1670 (3)	0.48205 (16)	0.38070 (8)	0.0233 (3)	
H17	0.1203	0.5659	0.4111	0.028*	
C18	0.0507 (3)	0.38732 (16)	0.39210 (9)	0.0238 (3)	
H18	−0.0756	0.4055	0.4316	0.029*	
C19	0.1165 (2)	0.26597 (16)	0.34642 (8)	0.0203 (3)	
H19	0.0329	0.2030	0.3542	0.024*	
C20	0.6813 (2)	−0.09205 (14)	0.33211 (8)	0.0163 (3)	
C21	0.5361 (2)	−0.17302 (15)	0.45788 (8)	0.0199 (3)	
H21A	0.6348	−0.1461	0.4955	0.024*	
H21B	0.6222	−0.2754	0.4458	0.024*	
C22	0.3031 (3)	−0.15439 (17)	0.49923 (9)	0.0251 (3)	
H22A	0.3301	−0.2167	0.5518	0.030*	
H22B	0.2069	−0.1814	0.4616	0.030*	
H22C	0.2199	−0.0529	0.5113	0.030*	
H1	1.081 (3)	−0.160 (2)	0.2506 (12)	0.037 (5)*	
H2	1.111 (3)	−0.118 (2)	0.1561 (12)	0.044 (5)*	

### Atomic displacement parameters (Å^2^)

**Table:**

	*U*^11^	*U*^22^	*U*^33^	*U*^12^	*U*^13^	*U*^23^
Cl1	0.0303 (2)	0.01992 (19)	0.0328 (2)	−0.00981 (15)	−0.00537 (15)	−0.00406 (15)
O1	0.0141 (5)	0.0219 (5)	0.0218 (5)	−0.0057 (4)	0.0003 (4)	0.0018 (4)
O2	0.0216 (5)	0.0269 (6)	0.0188 (5)	−0.0069 (4)	0.0051 (4)	−0.0046 (4)
O3	0.0142 (5)	0.0194 (5)	0.0159 (5)	−0.0043 (4)	0.0011 (4)	−0.0011 (4)
O4	0.0178 (5)	0.0202 (5)	0.0229 (5)	−0.0005 (4)	−0.0025 (4)	0.0027 (4)
O5	0.0171 (5)	0.0179 (5)	0.0163 (5)	−0.0037 (4)	−0.0009 (4)	0.0048 (4)
N1	0.0145 (6)	0.0204 (6)	0.0194 (6)	−0.0033 (5)	0.0018 (5)	−0.0030 (5)
C1	0.0169 (7)	0.0139 (6)	0.0175 (6)	−0.0076 (5)	−0.0007 (5)	−0.0012 (5)
C2	0.0184 (7)	0.0141 (6)	0.0174 (7)	−0.0075 (5)	−0.0018 (5)	−0.0013 (5)
C3	0.0194 (7)	0.0183 (7)	0.0215 (7)	−0.0082 (6)	−0.0015 (5)	−0.0010 (6)
C4	0.0229 (7)	0.0205 (7)	0.0227 (7)	−0.0072 (6)	−0.0077 (6)	0.0006 (6)
C5	0.0305 (8)	0.0221 (7)	0.0168 (7)	−0.0110 (6)	−0.0037 (6)	0.0003 (6)
C6	0.0247 (8)	0.0211 (7)	0.0162 (7)	−0.0099 (6)	0.0006 (5)	−0.0020 (6)
C7	0.0207 (7)	0.0147 (6)	0.0168 (6)	−0.0086 (5)	−0.0009 (5)	−0.0029 (5)
C8	0.0197 (7)	0.0151 (6)	0.0171 (6)	−0.0090 (5)	0.0018 (5)	−0.0043 (5)
C9	0.0161 (7)	0.0137 (6)	0.0170 (6)	−0.0058 (5)	−0.0007 (5)	−0.0025 (5)
C10	0.0166 (7)	0.0125 (6)	0.0156 (6)	−0.0062 (5)	−0.0001 (5)	−0.0012 (5)
C11	0.0147 (6)	0.0144 (6)	0.0148 (6)	−0.0056 (5)	−0.0005 (5)	0.0015 (5)
C12	0.0145 (6)	0.0135 (6)	0.0172 (6)	−0.0036 (5)	−0.0005 (5)	−0.0015 (5)
C13	0.0169 (7)	0.0128 (6)	0.0175 (6)	−0.0056 (5)	−0.0024 (5)	−0.0021 (5)
C14	0.0132 (6)	0.0136 (6)	0.0121 (6)	−0.0004 (5)	−0.0020 (5)	0.0016 (5)
C15	0.0140 (6)	0.0165 (7)	0.0123 (6)	−0.0019 (5)	−0.0005 (5)	0.0003 (5)
C16	0.0217 (7)	0.0163 (7)	0.0163 (6)	−0.0040 (6)	−0.0060 (5)	0.0015 (5)
C17	0.0261 (8)	0.0181 (7)	0.0157 (7)	0.0032 (6)	−0.0041 (5)	−0.0027 (5)
C18	0.0205 (7)	0.0248 (8)	0.0155 (7)	0.0020 (6)	0.0035 (5)	0.0005 (6)
C19	0.0181 (7)	0.0217 (7)	0.0172 (7)	−0.0042 (6)	0.0005 (5)	0.0031 (5)
C20	0.0162 (7)	0.0125 (6)	0.0186 (7)	−0.0034 (5)	−0.0002 (5)	−0.0027 (5)
C21	0.0222 (7)	0.0181 (7)	0.0152 (6)	−0.0042 (6)	−0.0033 (5)	0.0047 (5)
C22	0.0244 (8)	0.0299 (8)	0.0195 (7)	−0.0102 (6)	−0.0016 (6)	0.0058 (6)

### Geometric parameters (Å, °)

**Table:**

Cl1—C16	1.7479 (15)	C8—C9	1.4899 (18)
O1—C1	1.2177 (16)	C9—C10	1.3457 (18)
O2—C8	1.2231 (16)	C10—C11	1.5089 (17)
O3—C9	1.3584 (16)	C11—C12	1.5194 (18)
O3—C13	1.3751 (15)	C11—C14	1.5303 (19)
O4—C20	1.2274 (16)	C11—H11	1.0000
O5—C20	1.3492 (16)	C12—C13	1.3635 (18)
O5—C21	1.4549 (15)	C12—C20	1.4508 (18)
N1—C13	1.3372 (17)	C14—C19	1.3938 (18)
N1—H1	0.898 (18)	C14—C15	1.3959 (19)
N1—H2	0.880 (19)	C15—C16	1.3881 (19)
C1—C10	1.4834 (18)	C15—H15	0.9500
C1—C2	1.5002 (18)	C16—C17	1.387 (2)
C2—C3	1.3879 (19)	C17—C18	1.384 (2)
C2—C7	1.3959 (19)	C17—H17	0.9500
C3—C4	1.3963 (19)	C18—C19	1.390 (2)
C3—H3	0.9500	C18—H18	0.9500
C4—C5	1.379 (2)	C19—H19	0.9500
C4—H4	0.9500	C21—C22	1.498 (2)
C5—C6	1.386 (2)	C21—H21A	0.9900
C5—H5	0.9500	C21—H21B	0.9900
C6—C7	1.3984 (18)	C22—H22A	0.9800
C6—H6	0.9500	C22—H22B	0.9800
C7—C8	1.4739 (19)	C22—H22C	0.9800
			
C9—O3—C13	118.10 (10)	C14—C11—H11	108.1
C20—O5—C21	115.05 (10)	C13—C12—C20	117.77 (11)
C13—N1—H1	119.2 (11)	C13—C12—C11	122.20 (11)
C13—N1—H2	115.0 (12)	C20—C12—C11	120.02 (11)
H1—N1—H2	121.7 (17)	N1—C13—C12	128.18 (12)
O1—C1—C10	120.28 (11)	N1—C13—O3	109.49 (11)
O1—C1—C2	121.46 (11)	C12—C13—O3	122.33 (11)
C10—C1—C2	118.25 (11)	C19—C14—C15	119.25 (12)
C3—C2—C7	119.80 (12)	C19—C14—C11	120.14 (12)
C3—C2—C1	119.51 (12)	C15—C14—C11	120.59 (11)
C7—C2—C1	120.68 (11)	C16—C15—C14	119.13 (12)
C2—C3—C4	119.58 (13)	C16—C15—H15	120.4
C2—C3—H3	120.2	C14—C15—H15	120.4
C4—C3—H3	120.2	C17—C16—C15	122.12 (14)
C5—C4—C3	120.69 (13)	C17—C16—Cl1	118.47 (11)
C5—C4—H4	119.7	C15—C16—Cl1	119.39 (11)
C3—C4—H4	119.7	C18—C17—C16	118.20 (13)
C4—C5—C6	120.06 (13)	C18—C17—H17	120.9
C4—C5—H5	120.0	C16—C17—H17	120.9
C6—C5—H5	120.0	C17—C18—C19	120.85 (13)
C5—C6—C7	119.77 (13)	C17—C18—H18	119.6
C5—C6—H6	120.1	C19—C18—H18	119.6
C7—C6—H6	120.1	C18—C19—C14	120.40 (14)
C2—C7—C6	120.06 (12)	C18—C19—H19	119.8
C2—C7—C8	120.43 (12)	C14—C19—H19	119.8
C6—C7—C8	119.50 (12)	O4—C20—O5	121.56 (12)
O2—C8—C7	122.97 (12)	O4—C20—C12	125.79 (12)
O2—C8—C9	120.19 (12)	O5—C20—C12	112.64 (11)
C7—C8—C9	116.84 (11)	O5—C21—C22	107.88 (11)
C10—C9—O3	124.60 (12)	O5—C21—H21A	110.1
C10—C9—C8	124.01 (12)	C22—C21—H21A	110.1
O3—C9—C8	111.36 (11)	O5—C21—H21B	110.1
C9—C10—C1	119.43 (11)	C22—C21—H21B	110.1
C9—C10—C11	121.75 (12)	H21A—C21—H21B	108.4
C1—C10—C11	118.82 (11)	C21—C22—H22A	109.5
C10—C11—C12	109.30 (10)	C21—C22—H22B	109.5
C10—C11—C14	110.13 (10)	H22A—C22—H22B	109.5
C12—C11—C14	112.83 (11)	C21—C22—H22C	109.5
C10—C11—H11	108.1	H22A—C22—H22C	109.5
C12—C11—H11	108.1	H22B—C22—H22C	109.5
			
O1—C1—C2—C3	2.5 (2)	C1—C10—C11—C12	168.17 (11)
C10—C1—C2—C3	−176.36 (12)	C9—C10—C11—C14	113.41 (14)
O1—C1—C2—C7	−178.39 (13)	C1—C10—C11—C14	−67.34 (15)
C10—C1—C2—C7	2.70 (19)	C10—C11—C12—C13	14.24 (18)
C7—C2—C3—C4	−0.8 (2)	C14—C11—C12—C13	−108.66 (14)
C1—C2—C3—C4	178.23 (12)	C10—C11—C12—C20	−165.61 (11)
C2—C3—C4—C5	−0.7 (2)	C14—C11—C12—C20	71.50 (15)
C3—C4—C5—C6	1.6 (2)	C20—C12—C13—N1	−7.3 (2)
C4—C5—C6—C7	−1.0 (2)	C11—C12—C13—N1	172.81 (13)
C3—C2—C7—C6	1.5 (2)	C20—C12—C13—O3	172.24 (11)
C1—C2—C7—C6	−177.59 (12)	C11—C12—C13—O3	−7.6 (2)
C3—C2—C7—C8	−178.63 (12)	C9—O3—C13—N1	175.65 (11)
C1—C2—C7—C8	2.31 (19)	C9—O3—C13—C12	−4.00 (18)
C5—C6—C7—C2	−0.6 (2)	C10—C11—C14—C19	118.09 (13)
C5—C6—C7—C8	179.53 (13)	C12—C11—C14—C19	−119.49 (13)
C2—C7—C8—O2	173.40 (13)	C10—C11—C14—C15	−60.43 (15)
C6—C7—C8—O2	−6.7 (2)	C12—C11—C14—C15	62.00 (15)
C2—C7—C8—C9	−6.16 (18)	C19—C14—C15—C16	−2.01 (18)
C6—C7—C8—C9	173.74 (12)	C11—C14—C15—C16	176.52 (11)
C13—O3—C9—C10	7.43 (19)	C14—C15—C16—C17	2.07 (19)
C13—O3—C9—C8	−170.55 (10)	C14—C15—C16—Cl1	−176.00 (9)
O2—C8—C9—C10	−174.20 (13)	C15—C16—C17—C18	−0.47 (19)
C7—C8—C9—C10	5.37 (19)	Cl1—C16—C17—C18	177.61 (10)
O2—C8—C9—O3	3.80 (18)	C16—C17—C18—C19	−1.2 (2)
C7—C8—C9—O3	−176.63 (11)	C17—C18—C19—C14	1.2 (2)
O3—C9—C10—C1	−178.19 (12)	C15—C14—C19—C18	0.43 (19)
C8—C9—C10—C1	−0.5 (2)	C11—C14—C19—C18	−178.10 (12)
O3—C9—C10—C11	1.1 (2)	C21—O5—C20—O4	−2.29 (19)
C8—C9—C10—C11	178.79 (12)	C21—O5—C20—C12	176.26 (11)
O1—C1—C10—C9	177.45 (12)	C13—C12—C20—O4	6.9 (2)
C2—C1—C10—C9	−3.64 (19)	C11—C12—C20—O4	−173.26 (13)
O1—C1—C10—C11	−1.82 (19)	C13—C12—C20—O5	−171.59 (12)
C2—C1—C10—C11	177.09 (11)	C11—C12—C20—O5	8.26 (17)
C9—C10—C11—C12	−11.08 (17)	C20—O5—C21—C22	−175.25 (12)

### Hydrogen-bond geometry (Å, °)

**Table:**

*D*—H···*A*	*D*—H	H···*A*	*D*···*A*	*D*—H···*A*
N1—H1···O4	0.898 (18)	2.049 (18)	2.6827 (17)	126.5 (15)
N1—H2···O2^i^	0.880 (19)	2.12 (2)	2.9913 (17)	170.2 (18)

Symmetry codes: (i) −*x*+2, −*y*, −*z*.
